# Supplementary material for: Exploring the motivations of female community health volunteers in primary healthcare provision in rural Nepal: A qualitative study
Source: PLOS Glob Public Health. 2024 Aug 1;4(8):e0003428. doi: 10.1371/journal.pgph.0003428 (PMC11293747; doi:10.1371/journal.pgph.0003428)
Supplement: S1 Checklist — (DOCX) [file pgph.0003428.s001.docx]

Inclusivity in global research

PLOS’ policy on inclusivity in global research aims to improve transparency in the reporting of research performed outside of researchers’ own country or community and ensures that PLOS publications reporting global research adhere to high standards for research ethics and authorship. Authors of relevant research articles may be asked to complete the questionnaire below, which outlines ethical, cultural, and scientific considerations specific to inclusivity in global research. This questionnaire may be requested when researchers have travelled to a different country to conduct research, if research uses samples collected in another country, research with Indigenous populations or their lands, or if research is on cultural artefacts. Researchers travelling to another country solely to use laboratory equipment will not normally be required to complete the questionnaire. However, the questionnaire can be requested at the journal’s discretion for any submission – if you have been requested to complete this questionnaire by the PLOS journal you submitted to, please do so.

Please complete the questionnaire below and include this as a Supporting Information file with your manuscript. Note that if your paper is accepted for publication, this checklist will be published with your article in the supporting information files. Please ensure that you reference the checklist in the main body of your manuscript. We suggest adding a subsection ‘Inclusivity in global research’ to your Methods section and adding the following sentence: “Additional information regarding the ethical, cultural, and scientific considerations specific to inclusivity in global research is included in the Supporting Information (S1 Checklist)”

The questions have been designed to be applicable to a wide range of study types, and there are subsections for both human subjects research and non-human subjects research. If any of the questions are not relevant to your research please mark them as “N/A” as appropriate.

**Ethical considerations, permits and authorship**

*This section is applicable to all research types.*

Provide details as to who granted permissions and/or consent for the study to take place in the Methods section of your manuscript. This should include the names of **all** ethics boards, governmental organizations, community leaders or other bodies that provided approval for the study. If individuals provided approval refer to these people by their role or title but do not list their name(s).

This is reported in an ethics statement on p10. of the manuscript. To confirm, ethical approval for the study was received from the Nepal Health Research Council Ethical Review Board in 2013 (Registration number 32/2013).

If there were any deviations from the study protocol after approval was obtained please provide details of these changes in the Methods section of your manuscript.

Reported on page number 10: No deviations were made from the study protocol after ethics approval.

Did this study involve local collaborators that are residents of the country where the research was conducted or members of the community studied? If you do not have any authors from said communities, please provide an explanation for this below.

This study involved members of two communities from Nepal. The study does not have any authors from these two communities, however, the research is grounded in the views of the female community health volunteers (FCHVs) who were involved and they are directly quoted on the paper. The first author is from one of the study sites (Dhading) in Nepal, who is aware of local norms and can speak local language (Nepali). She planned the study, collected the data, analysed them with support from other co-authors in the UK.

Everyone listed as an author should meet PLOS’ criteria for authorship and all individuals who meet these criteria should be included in the author byline, rather than the acknowledgements. For further information please see the journal’s Authorship Policy.

**Human subjects research (e.g. health research, medical research, cross-cultural psychology)**

Did you obtain written informed consent from a representative of the local community or region before the research took place? How did you establish who speaks for the community? Details of written informed

consent obtained from study participants should be reported separately in the Methods section of your manuscript.

Written informed consent was obtained from Nepal Health Research Council before the research took place as the main ethics body for representing communities as a whole in the country. As this study aimed to explore experiential factors influencing female community health volunteers’ (FCHVs) motivations in two villages of Nepal, the first author met with local community representatives, including district health workers, local health workers and elected representatives in the study communities to talk about the research and help identify eligible FCHVs who could be approached to see if they were interested to take part in the study. In practice, we ensured that the FCHVs in this study represented ethnically diverse population. For example, Muslim and Madhesi population in Terai, and Chepang in the hill village. Volunteers representing diverse ethnic groups, with varying levels of work experience and education were included as shown in Table 2.

How did members of the local community provide input on the aims of the research investigation, its methodology, and its anticipated outcome(s)?

I spoke to members of local community, including representatives of volunteers, mothers, and health workers from study districts before I started my research. They were welcoming and positive about the aims of the research investigation. They helped me to locate marginalised populations, including FCHV who represented the groups.

The principal investigator also involved local people (one each in the study area) to assist with the project. This was useful to reach people who lived far away from the local health centre and enabled rapport building among local participants.

In terms of anticipated outcomes, the first author had explicitly mentioned that it was a research project and might not bring any immediate changes in study participants’ lives.

When engaging with the local community, how did you ensure that the informed consent documents and other materials could be understood by local stakeholders?

The informed consent documents and information sheet were translated into Nepali, so that the key stakeholders could read it in local language. For illiterate participants , the first author read out the consent form and obtained their written signature (some participants could not read the form but were able and content to sign their names.

Will the findings of the research be made available in an understandable format to stakeholders in the community where the study was conducted (e.g. via a presentation, summary report, copies of publications, etc.)? Please provide details of how this will be achieved.

Yes, the findings of the research will be made available in an understandable format to stakeholders in the community where the study was conducted. Lay summary of the research will be written and translated into Nepali and will be shared and presented in person during the  authors’ next visit to the study sites.

**Non-human subjects research using specimens/ animals collected as part of the study, or those housed in archival collections. Examples include archaeology, paleontology, botany and zoology.**

Did the permission you obtained from a local authority to perform the study include an agreement on access to outputs and benefit sharing? This may include procedures to enable fair distribution of the benefits and resources arising from the research performed. Please include any details of Prior Informed Consent and Benefit Sharing Agreements obtained. These may be required by field-specific regulations, for example the Convention on Biological Diversity (CBD) and the associated Nagoya Protocol.

N/A

If the material used in your study was imported, please A) provide the year it was imported and B) indicate whether permits were obtained to import/export the materials used, C) provide details of any permits obtained. If this information is not available, please indicate this.

N/A

If you used archival specimens, please state how the material used in your study was acquired by the institute it is held in and provide details of any permits obtained for the original excavations/ sample collection. If this information is not available, please indicate this.

N/A

How was the potential cultural significance of the materials collected in your study to local communities considered in your research design? Were Indigenous peoples and/or local researchers and institutions involved with archaeological excavations / collection of specimens? If so, please provide a description of their involvement.

N/A

If your manuscript includes photographs of human remains please indicate whether authors obtained permission from descendants or affiliated cultural communities to do so.

N/A
