# Supplementary material for: Exploring the motivations of female community health volunteers in primary healthcare provision in rural Nepal: A qualitative study
Source: PLOS Glob Public Health. 2024 Aug 1;4(8):e0003428. doi: 10.1371/journal.pgph.0003428 (PMC11293747; doi:10.1371/journal.pgph.0003428)
Supplement: Attachment — Submitted filename: Response to Reviewers.docx [file pgph.0003428.s003.docx]

**Response to Reviewers**

We are grateful to the 3 reviewers for their valuable comments and feedback to improve the manuscript. We really appreciate their time. We have revised our manuscript to reflect the reviewers’ suggestions and comments. Below is a point-by-point response to each reviewer describing exactly what amendments have been made to the manuscript text and where these can be viewed (section, line number and page number).

Reviewer #1: This paper examines the factors influencing the motivations of Female Community Health Volunteers (FCHVs) in rural Nepal, which reveal truly interesting and significant issues regarding primary health care delivery. Here are several points that the authors can improve before publication:

1. The authors mentioned gender inequality and the relevant hierarchy. How does gender inequality or discrimination influence the FCHVs' motivation? Is it also a factor of motivation (or disincentives)?

Thank you for your valuable suggestion. We have included the following explanation in the discussion section (page 29, line number 625-633).We also found that regardless of a volunteers’ age, they wanted to retain their position as a volunteer despite financial challenges, given how they felt it provided women with greater social status and was a route to self-empowerment . For example, in our findings above, and in our previous work on this topic, we highlight how FCHVs appreciated the opportunities to learn new knowledge and skills, and valued travelling outside their houses, meeting new people, and gaining respect from health workers for the work they undertook, which might not be possible for most women in villages [25]. Such empowering experiences can be crucial for women in societies with low social status.

2. The authors analyzed the qualitative data using thematic analysis and generated themes at individual, organizational, and community levels, it would be more helpful and clearer to understand if the authors could illustrate and summarize the different themes/subthemes using a structural figure or framework in the result part.

Thank you, a new Figure has been developed to respond to this point and inserted in Section Results on page 17 as shown below.

3. The data was collected in 2014, whereas the authors mentioned the FCHV programme was revised in 2010 and 2019, I wonder whether there can be any factors that may alter the motivation so far due to the policy revision. Despite the authors mentioning the number of FCHVs and their roles remain similar, a brief summary of the policy content change (or what does not change) would be helpful to justify the potential application of results to current circumstances.

Thank you. A new paragraph has been added in Section Introduction on Page 5-6 setting out that

the FCHV strategy was subsequently revised three times in 1990, 1992 and 2003, before the formulation of a new FCHV strategy in 2010 [29-31]. When the programme began, FCHVs were provided with 100 Nepalese rupees (NRs) per month and a training allowance of NRs 250. However, monetary incentives were withdrawn in 1990. In 1992, the training allowance was reinstated, but no other incentives for volunteers until 2010, when the new FCHV strategy (2010) emphasised provision of several non-monetary incentives, such as a celebration of FCHV day and provision of uniform [30]. AFCHV fund (NRs 50, 000) was also created at a village level to support volunteers’ livelihoods through a loan (29), but a survey of volunteers reported that only 66% of FCHVs had heard of the fund and only 51% were members of the fund [31]. The FCHV strategy (2010) was revised again in 2019 to include incentives for FCHVs to attend work-related meetings according to government provision, included provision of a letter of honour and a sum of NRs 10, 000 for retiring FCHVs above 60 years old [29].

4. The questionnaire of semi-structured interview and the list of focus group questions are recommended to be attached in the supplementary appendix.

These have been attached in the supplementary appendix (S2 Interviews and focus groups guide).

5. It might be out of the scope of this study, but I am interested in whether the qualitative results are systematically different between the two selected regions, as the authors emphasized that the two rural settings were selected based on different characteristics.

In terms of financial incentives, study participants in both regions emphasised the necessity of compensating volunteers for their work as reported in this manuscript. However, key information about the differences in the two study regions have been added in Section Results on page 23, line number 484-486.

The available public health system support in terms of medical supplies and regular supervision of FCHVs were systematically different in the two selected regions (hill villages and the Terai), which has been reported in our earlier study [25].

Minor:

1. The numbering of the result part is messed up, and the format of headings and main texts is not consistent (e.g. main texts before and after 4.2 are in different line spacing), please correct them.

Yes, thank you, we have corrected them.

2. For the organizational-level and community-level factors, only one factor was analyzed in detail

(Bureaucratisation and community perception), the extra subtitles seem redundant.

Yes, thank you, we have removed extra subtitles.

Reviewer #2: Thanks for inviting me to review this research article “Exploring the motivations of female community health volunteers in primary healthcare provision in rural Nepal: a qualitative study”. Based on the qualitative investigation and analysis, FCHVs’ motivations to volunteer in rural Nepal were affected in several unique ways. After evaluating this study, I recommend it for publication in this Journal, with revisions. Below are my comments and suggestions.

1. In the method section, authors need to describe the settings the way such as the meeting rooms/open spaces where the interviews/focus groups were conducted.

Thank you. The following information has been included in Section Method, Data collection on page 11, line number 267-272:

Interviews and focus groups were conducted using a semi-structured thematic interview guide (S2 interviews and focus groups guide) and most were conducted in participants’ homes, although some individual interviews and group discussions were held in a meeting room at health centres, or local cafes as per participants’ availability and preference. For example, the first author conducted a focus group discussion and also conducted an individual interview with a volunteer from a remote area after their monthly meeting at the local health centre.

2. In the method section, compared to the volunteer participation, can authors provide more detail of the paid participation in this study such as the amount of payment or covered costs?

The following information has been included in Section Method, ‘data collection’ on page 11, 12:

In terms of research-related payments, 15 FCHVs who had to commute to participate in interviews were paid NRs 500 (US $3.46) to cover their expenses. No other incentives were provided to the participants to take part in the study. However, when possible, refreshments (tea, biscuits, snacks, cold drinks) were arranged after the interview/group discussions.

3. The authors present the socio-demographic characteristics of FCHVs (Table 2), age is also an important factor but it hasn’t been reported. For any unreported data in the tables, please indicate it as unknown.

Thank you, a column with age of volunteers have been added to Table 1 on page 13.

There was no unreported data in the tables. Ditto sign [″] was meant to say same as above, but we removed this to avoid confusion and replaced with exact characteristics of the study participants in the tables (1 and 2) on page 13 and 15.

4. In every narrative extract, can authors briefly describe the narrators (e.g. socio-demographics) to facilitate reading?

Thank you. We have amended the identifiers used in relation to each narrative extract. Each quote has the following identifiers (age range, years of education; exp= years of experience in the post; and distance= walking distance to health centres in minutes/hrs otherwise stated). – we have explained this identification system in Section Method on page 16, line number 331-333 of the manuscript.

5. In the discussion section, more discussion of potential integrated solutions across three levels (individual, organization, and community) would be sound.

Thank you for this useful suggestion. We have added in more detail into the discussion about the need for integrated action across the health system with examples of what would be needed at different levels, based on the findings from our research. Please see page 32, line number 696-709

This however, has financial implications for achieving health related goals (SDG3), suggesting that more financial investment for FCHVs is needed for integrated action across three levels (individual, organisational and community) . For example, motivating volunteers at individual level will require national policy change, not only to re-enact the financial incentives that were present in the FCHV scheme when it first began, but also to ensure that the incentives match their workload and there is a greater recognition of the value of FCHVs in national policy. At organisational level, it will require the implementation of procedures and practices to ensure that FCHVs get all the financial incentives that they are due and in a timely way and also a programme of cultural change to improve the way that paid health workers value and interact with FCHVs to ensure that their relationships become grounded in and engender respect. At community level, integrated action is needed to increase community awareness of FCHVs’ roles and their contribution to community health.

Reviewer #3: This paper by Panday et al. was a qualitative study exploring the motivations of female CHWs who are volunteers in delivering PHC services in rural Nepal. The authors conducted 31 semi-structured interviews and 3 focus groups among female CHWs including volunteers and paid workers in two districts of Nepal. Data was analyzed thematically and organized using the conceptual framework developed by Franco et al. Individual,

organizational, and community level results included: 1) volunteers’ commitment to supporting mothers and children; disincentives and opportunity costs of volunteering; 2) bureaucratisation of volunteer work; and 3)community perception of volunteers. The authors concluded more support is needed at all these levels, and financial investments are necessary.

The paper addresses a clear research gap. Strengths include a straightforward background section anddiscussion which highlighted the relevance of the study. The conclusions drawn are supported by the results,with minor areas for improvement, and the method and discussion sections need some more detail. The main weakness is a results section presented without the use of tables or figures. This makes it challenging to synthesize the findings. There are also several typographical errors requiring copyediting. Overall, I recommend this paper is accepted with revisions.

Major areas of improvement include:

1) The lack of tables or figures in the results section. I strongly suggest creating a table or figure which organizes the results by individual, organizational, and community levels. Within each of these levels the authors can include their subthemes and draw connections between them. An example is provided by Figure 1 in a paper on the contribution of FCHVs to maternity care in Nepal also by Panday et al.

Panday S, Bissell P, Van Teijlingen E, Simkhada P. The contribution of female community health volunteers (FCHVs) to maternity care in Nepal: a qualitative study. BMC health services research. 2017 Dec;17(1):1-1.

Thank you. We have produced a new figure to respond to this point and inserted in Section Results on page 17.

2) I suggest the authors summarize their three tables on participant information into just one table.

Thank you. We removed Table 1, but kept Table 2 and 3, as they provide different information: Table 1 is about characteristics of FCHVs and Table 2 is about health workers. Please see the changes in Section Method on page 13 and 15.

3) In the introduction the authors describe data was collected for this study nine years ago. They do a good job defending the continued relevance of their findings, but maybe they can include a line on how this paper makes a different contribution from their earlier paper, which draws from the same data.

Thank you. The following information has been added in Section Introduction on Page 7, line number 186-188):

Recent research in Nepal reveals that FCHVs experience issues such as workload and payment [25, 39, 40]. These are issues that we go on to highlight in this current paper, which therefore suggests the continued salience of our previous research [41].

4) Some more detail is needed in the reporting of the thematic analysis in the methods section. For example, Braun and Clarke suggest creating initial thematic maps. Perhaps a codebook could be included as an appendix too.

Thank you. The following information has been added in Section Method on page 15, line number 316 and line number 324.

EvT coded four interviews and a group discussion transcripts in English independently.

Common themes across the data set were developed in NVivo at three levels.

The PI tried to open NVivo 12 file in which coding was done, but could not do so due to technical error at this instance.

5) The discussion section occasionally needs greater detail when connecting with the broader literature. For example, on page 23 the authors write “These findings are consistent with CHW literature from sub-Saharan Africa and South Asia (51, 52)”. Please expand what are the findings from these other studies if relevant.

Thank you. We have included some greater detail when connecting with broader literature. For example, the following information has been added in Section Discussion on Page 28-29:

For example, in a study conducted in Bangladesh, India, Kenya, Malawi and Nigeria, involving thirty-two focus group discussions with 361 individuals and 116 key informant interviews with CHWs, health workers and managers, it was found that CHWs consistently expressed a need for appropriate and consistent compensation for their work [54]. Similarly, another study of CHWs that also included their supervisors and high-level officials (n=95) within Global Polio Eradication Initiative in India, Nepal, Pakistan, Ethiopia and Rwanda showed that when CHWs were provided with some financial compensation, this was perceived to be low and exploitative of CHWs’ work [55].

6) Since the authors conclude more financial investment is needed to support FCHVs, I strongly suggest including more extracts explicitly identifying this (presently only extracts 5, 6, 8, 9, 16 mention salary/finances).

Thank you, we have emphasised the unpaid nature of FCHVs work throughout the findings and have clarified, for example, the importance of lack of coverage out of pocket expenditures. We would like to highlight that other included quotes/extracts, e.g. the quotes on p.23-26 (previously extracts 12, 14 and 17) also relate to finances.

Copyediting is needed for this paper. Minor areas of improvement include:

Thank you for the copyediting suggestions – we have been through the whole paper and have made changes relating to all the below.

1) Consistency between using the words “female” or “women”.

2) Numbers nine and below should be spelled out.

3) The format the date is presented in line 213 may be ambiguous.

4) Replace the apostrophe symbol in the tables.

5) Use oxford commas.

6) Fix punctuation such as double periods and spaces.

My only other comment is the authors write “informed consent was obtained from most of the participants”. I am confused how data was collected then for participants who did not provide consent.

Apologies for this error in our writing, it has been corrected as follows:

Informed written consent was obtained from all the participants included in the study and responses were anonymised (Method, page 10).
